# The Unlucky Blow

**Published:** 1870-08

**Authors:** 


### THE UNLUCKY BLOW.

A BRIGHT little boy of six years, ran to his father one day to ask him for a penny for a beggar. The father was very busy and the little boy very importunate; in a moment of irritation, the father hit him on the head; he staggered and fell, and from that moment until the present day, embracing twelve long years, the child became deaf, and dumb, and blind, and now utters inarticulate words which are horrible to hear, and which only the stricken mother can understand.

Children have been made deaf for life, by being "boxed" on the ear, rupturing the "drum," which is as thin as writing paper.

A school-teacher aimed to give a derelict scholar a tap on the head with his pen-knife; the blade flew out, the point entered the brain at the joinings of the skull, and instant death ensued. Let parents and teachers be warned by these incidents never to strike at an unresisting child in anger; it is a cowardice, a brutality, and a crime.

